# Supplementary material for: Intra-aneurysmal pressure changes during stent-assisted coiling
Source: PLoS One. 2020 Jun 4;15(6):e0233981. doi: 10.1371/journal.pone.0233981 (PMC7272096; doi:10.1371/journal.pone.0233981)
Supplement: S1 File — (PDF) [file pone.0233981.s001.pdf]

site	level	region	stent_type	stent_cell	stent	RRIASP1
LACA	A1	ACOA	NA	NA	0	68
ACOA	NA	ACOA	NA	NA	0	.
BA	NA	BA	NA	NA	0	79
LICA	C6/7	LICA	NA	NA	0	65
LICA	C5	LICA	NA	NA	0	74
LICA	C6	LICA	NA	NA	0	120
LICA	C5	LICA	NA	NA	0	76
LICA	C5	LICA	NA	NA	0	.
MCA	M1/M2	MCA	NA	NA	0	80
RMCA	M1/M2	MCA	NA	NA	0	.
RICA	C6	RICA	NA	NA	0	64
RICA	C6	RICA	NA	NA	0	76
RICA	C6	RICA	NA	NA	0	49
LACA	A2	ACOA	LVIS Jr	1,5	1	70
BA	NA	BA	Barrell	.	1	46
BA	NA	BA	LVIS Jr	1,5	1	77
BA	NA	BA	LVIS Jr	1,5	1	84
BA	NA	BA	LVIS Jr	1,5	1	73
BA	NA	BA	LVIS Jr	1,5	1	71
LICA	C4/C5	LICA	LVIS	1	1	68
LICA	C5/6	LICA	LVIS	1	1	68
LICA	C5/6	LICA	LVIS	1	1	86
LICA	C5/6	LICA	LVIS	1	1	70
LICA	C5/6	LICA	Leo	0,7	1	61
LICA/MCA	C7/M1	LICA	LVIS	1	1	77
LICA	C5	LICA	Leo	0,63	1	68
LICA	C6	LICA	Leo	0,7	1	.
LICA	C6	LICA	Leo	0,63	1	57
LICA	C4	LICA	LVIS	1	1	69
LICA	C5/6	LICA	Leo	0,63	1	87
LICA	C6	LICA	Leo	0,63	1	79
RICA	C5/6	RICA	LVIS	1	1	88
RICA	C5/6	RICA	LVIS	1	1	75
RICA	C5	RICA	Leo	0,63	1	63
RICA	C6/7	RICA	Leo	0,63	1	64
RICA	C5	RICA	LVIS	1	1	91
RICA	C7	RICA	LVIS	1	1	58
RICA	C4	RICA	LVIS	1	1	87
RICA	C5/6	RICA	Leo	0,63	1	71
RICA	C2	RICA	Leo	0,7	1	65
RICA	C5	RICA	Leo	0,63	1	63
RICA	C5/6	RICA	LVIS	0,9	1	62
RICA	C6	RICA	Leo	0,63	1	70
RICA	C7	RICA	LVIS Jr	1,5	1	109
RICA	C5/6	RICA	Leo	0,63	1	55

RRIADP1	RRIAMAP1	RRsystSP1	RRsystDP1	RRsystMAP1	HR1	RRIASP2
66	66,6666667	102	52	68,6666667	75	66
.	.	.	.	.	.	67
70	73	117	68	84,3333333	79	80
60	61,6666667	120	70	86,6666667	78	66
60	64,6666667	106	70	82	70	75
112	114,666667	160	100	120	85	123
66	69,3333333	113	65	81	61	.
.	.	.	.	.	.	86
72	74,6666667	119	70	86,3333333	53	81
.	.	.	.	.	.	52
54	57,3333333	98	57	70,6666667	81	64
71	72,6666667	98	67	77,3333333	67	70
44	45,6666667	94	47	62,6666667	66	.
66	67,3333333	98	63	74,6666667	70	74
40	42	102	64	76,6666667	72	.
73	74,3333333	104	61	75,3333333	60	80
78	80	112	69	83,3333333	64	.
65	67,6666667	72	66	68	71	72
58	62,3333333	104	51	68,6666667	60	72
59	62	105	62	76,3333333	56	79
59	62	105	62	76,3333333	56	.
75	78,6666667	122	66	84,6666667	52	81
60	63,3333333	108	59	75,3333333	63	70
59	59,6666667	101	47	65	54	.
72	73,6666667	110	72	84,6666667	72	77
59	62	98	61	73,3333333	66	64
.	.	.	.	.	.	.
46	49,6666667	102	58	72,6666667	74	55
60	63	80	50	60	60	74
71	76,3333333	106	63	77,3333333	69	80
67	71	113	68	83	70	79
85	86	124	89	100,666667	81	79
67	69,6666667	101	67	78,3333333	67	76
56	58,3333333	97	53	67,6666667	69	64
54	57,3333333	110	56	74	63	64
75	80,3333333	103	64	77	56	84
55	56	99	58	71,6666667	75	65
71	76,3333333	95	56	69	58	83
70	70,3333333	131	64	86,3333333	73	.
58	60,3333333	94	58	70	62	61
62	62,3333333	95	55	68,3333333	58	64
51	54,6666667	85	50	61,6666667	51	63
66	67,3333333	107	64	78,3333333	64	67
100	103	138	80	99,3333333	56	.
48	50,3333333	85	50	61,6666667	70	55

RRIADP2	RRIAMAP2	RRsystSP2	RRsystDP2	RRsystMAP2	HR2	RRIASP3
64	64,6666667	100	51	67,3333333	73	.
61	63	107	74	85	68	.
75	76,6666667	117	68	84,3333333	89	.
60	62	120	70	86,6666667	78	.
63	67	95	60	71,6666667	70	.
116	118,333333	160	100	120	85	.
.	.	.	.	.	.	.
77	80	117	78	91	61	.
72	75	123	70	87,6666667	53	.
51	51,3333333	96	53	67,3333333	47	.
55	58	98	57	70,6666667	80	.
68	68,6666667	98	67	77,3333333	67	.
.	.	.	.	.	.	.
71	72	103	76	85	72	.
.	.	.	.	.	.	54
74	76	104	61	75,3333333	59	78
.	.	.	.	.	.	75
66	68	110	58	75,3333333	71	74
59	63,3333333	102	51	68	59	73
68	71,6666667	105	63	77	55	74
.	.	.	.	.	.	74
71	74,3333333	115	68	83,6666667	57	.
68	68,6666667	106	60	75,3333333	64	72
.	.	.	.	.	.	61
72	73,6666667	110	72	84,6666667	71	80
58	60	98	61	73,3333333	66	64
.	.	.	.	.	.	78
47	49,6666667	102	58	72,6666667	74	57
60	64,6666667	80	50	60	60	56
76	77,3333333	106	63	77,3333333	69	86
70	73	113	68	83	70	67
74	75,6666667	112	81	91,3333333	70	78
68	70,6666667	101	67	78,3333333	67	76
61	62	97	53	67,6666667	69	66
62	62,6666667	110	56	74	62	56
76	78,6666667	108	65	79,3333333	54	15
62	63	101	64	76,3333333	73	65
76	78,3333333	95	56	69	59	75
.	.	.	.	.	.	74
58	59	94	58	70	63	63
63	63,3333333	95	55	68,3333333	59	73
58	59,6666667	88	52	64	55	.
64	65	103	62	75,6666667	68	54
.	.	.	.	.	.	105
51	52,3333333	85	50	61,6666667	68	57

RRIADP3	RRIAMAP3	RRsystSP3	RRsystDP3	RRsystMAP3	HR3	RRIASP4
.	.	.	.	.	.	.
.	.	.	.	.	.	60
.	.	.	.	.	.	65
.	.	.	.	.	.	56
.	.	.	.	.	.	.
.	.	.	.	.	.	100
.	.	.	.	.	.	.
.	.	.	.	.	.	.
.	.	.	.	.	.	72
.	.	.	.	.	.	66
.	.	.	.	.	.	78
.	.	.	.	.	.	89
.	.	.	.	.	.	.
.	.	.	.	.	.	75
50	51,3333333	104	74	84	70	61
71	73,3333333	100	60	73,3333333	57	76
69	71	100	61	74	59	72
69	70,6666667	101	57	71,6666667	72	63
60	64,3333333	107	55	72,3333333	58	95
68	70	111	66	81	53	.
66	68,6666667	103	62	75,6666667	51	68
.	.		.	.		.
67	68,6666667	109	58	75	64	101
56	57,6666667	100	46	64	53	.
76	77,3333333	111	71	84,3333333	70	.
56	58,6666667	102	65	77,3333333	68	56
69	72	103	63	76,3333333	73	82
54	55	109	62	77,6666667	73	86
50	52	92	50	64	62	58
77	80	116	67	83,3333333	66	83
59	61,6666667	103	59	73,6666667	58	.
74	75,3333333	123	78	93	68	.
68	70,6666667	105	67	79,6666667	66	75
61	62,6666667	98	58	71,3333333	69	.
51	52,6666667	112	58	76	65	58
10	11,6666667	120	69	86	55	88
58	60,3333333	101	60	73,6666667	64	78
64	67,6666667	97	60	72,3333333	58	72
71	72	116	56	76	75	66
58	59,6666667	94	58	70	63	63
69	70,3333333	110	63	78,6666667	60	73
.	.	.	.	.	.	63
51	52	98	53	68	74	64
98	100,333333	143	80	101	56	.
53	54,3333333	88	48	61,3333333	70	54

RRIADP4	RRIAMAP4	RRsystSP4	RRsystDP4	RRsystMAP4	HR4	RRIASP5
.	.	.	.	.	.	.
53	55,3333333	93	62	72,3333333	63	56
64	64,3333333	141	63	89	84	84
50	52	102	60	74	75	.
.	.	.	.	.	.	.
82	88	111	87	95	84	103
.	.	.	.	.	.	.
.	.	.	.	.	.	.
66	68	109	66	80,3333333	51	79
65	65,3333333	100	54	69,3333333	48	51
71	73,3333333	104	61	75,3333333	78	.
86	87	123	82	95,6666667	63	97
.	.	.	.	.	.	.
70	71,6666667	102	65	77,3333333	72	.
56	57,6666667	109	74	85,6666667	69	66
71	72,6666667	99	58	71,6666667	57	.
63	66	94	61	72	55	73
60	61	96	54	68	68	70
75	81,6666667	137	64	88,3333333	52	92
.	.	.	.	.		75
68	68	98	61	73,3333333	51	75
.	.	.	.	.	.	.
51	67,6666667	110	56	74	65	98
.	.	.	.	.	.	61
.	.	.	.	.	.	.
52	53,3333333	103	63	76,3333333	73	63
77	78,6666667	101	65	77	77	79
83	84	118	72	87,3333333	107	.
52	54	118	66	83,3333333	57	59
74	77	106	64	78	63	84
.	.	.	.	.	.	.
.	.	.	.	.	.	.
68	70,3333333	104	65	78	65	71
.	.	.	.	.	.	.
56	56,6666667	120	62	81,3333333	64	73
78	81,3333333	120	73	88,6666667	54	94
69	72	123	74	90,3333333	72	86
64	66,6666667	102	58	72,6666667	67	71
61	62,6666667	98	46	63,3333333	68	68
58	59,6666667	94	58	70	62	63
70	71	110	71	84	61	44
58	59,6666667	87	51	63	55	64
62	62,6666667	97	56	69,6666667	66	64
.	.	.	.	.	.	.
49	50,6666667	94	53	66,6666667	73	60

RRIADP5	RRIAMAP5	RRsystSP5	RRsystDP5	RRsystMAP5	HR5
.	.	.	.	.	.
53	54	93	64	73,6666667	63
77	79,3333333	141	63	89	91
.	.	.	.	.	.
.	.	.	.	.	.
86	91,6666667	111	87	95	84
.	.	.	.	.	.
.	.	.	.	.	.
66	70,3333333	111	66	81	52
49	49,6666667	105	54	71	48
.	.	.	.	.	.
89	91,6666667	122	79	93,3333333	67
.	.	.	.	.	.
.	#VALUE!	.	.	#VALUE!	.
60	62	111	76	87,6666667	70
.	.	.	.	.	.
65	67,6666667	94	61	72	56
65	66,6666667	96	54	68	68
73	79,3333333	133	62	85,6666667	51
62	66,3333333	106	63	77,3333333	52
62	66,3333333	106	63	77,3333333	52
.	.	.	.	.	.
50	66	110	56	74	65
59	59,6666667	101	47	65	54
.	.	.	.	.	.
62	62,3333333	103	63	76,3333333	73
72	74,3333333	100	64	76	74
.	.	.	.	.	.
53	55	118	64	82	58
75	78	106	64	78	65
.	.	.	.	.	.
.	.	.	.	.	.
66	67,6666667	101	64	76,3333333	65
.	.	95	58	70,3333333	64
68	69,6666667	128	66	86,6666667	64
81	85,3333333	120	75	90	54
80	82	123	74	90,3333333	73
63	65,6666667	102	58	72,6666667	66
63	64,6666667	98	46	63,3333333	67
58	59,6666667	94	58	70	62
43	43,3333333	94	61	72	60
59	60,6666667	87	51	63	55
59	60,6666667	97	56	69,6666667	63
.	.	.	.	.	.
54	56	95	54	67,6666667	77

microcatheter distal diameter	packing_%	coil_vol	an_vol	Dmax	W
0,43	0	0	16,7	2,9	4,4
0,43	23,04	4,02	17,45	3,5	3,4
0,43	15,17	7,39	48,71	5,1	3,8
0,43	6,6	32,32	489,91	10,6	9,1
0,43	15,9	24,32	153	7,7	6,9
0,43	16,34	6,7	41	3	8,7
0,43	0	0	28,71	3,9	3,7
0,43	0	0	101,83	4,9	8,1
0,5	6,75	279,42	4139,01	23,9	14,7
0,43	40,32	9,93	24,63	4	4,9
0,43	9,36	216,16	2309,05	17,7	16,5
0,43	25,05	18,58	74,17	5,7	7,1
0,43	0	0	49,81	4,7	4,4
0,43	33,53	10,72	31,97	3,7	5
0,43	9,17	75,6	824,33	11,7	11,7
0,43	0	0	48,69	6,3	3,6
0,43	26	35,87	137,97	5,9	7,7
0,43	68,72	10,72	15,6	3,1	3,1
0,43	37,58	78,02	208,08	7,6	8,3
0,43	31,91	16,66	52,21	4,1	7,6
0,43	0	0	59,04	5,4	5,8
0,43	0	0	105,11	5,1	8,2
0,43	24,1	33,48	138,94	6,1	7,5
0,43	27,07	24,67	91,12	7,1	5,7
0,43	26,59	35,14	132,15	5,7	8,2
0,43	21,5	37,86	176,09	6	9,5
0,43	7,87	4,02	51,1	4	6,1
0,43	44,74	29,36	65,62	5,9	3,6
0,43	6,67	159,8	2395,25	17,6	22,8
0,43	21,53	129,81	602,81	10,4	12,3
0,43	24,81	44,06	177,58	7,3	10,1
0,43	33,14	36,25	109,38	6,2	6,4
0,43	21,49	88,49	411,86	7,6	11,5
0,43	24,76	7,05	28,47	3,9	4,1
0,43	14,67	12,91	88,01	5,8	6,9
0,43	10,28	8,92	86,78	5,3	5,9
0,43	25,67	40,51	157,84	6,3	8,7
0,43	26,01	17,78	68,37	4,9	6,5
0,43	37,21	15,41	68,37	5,2	3,9
0,43	,	,	,	.	.
0,43	44,42	9,12	20,53	3,5	4
0,43	46,51	44,1	94,82	5,3	6,7
0,43	61,57	30,57	49,65	4,9	4,3
0,43	6,23	168,9	2710,28	20,5	12,5
0,43	81,01	20,77	25,64	3,4	4

H	N	X1	X2	X3	Xn	delta
2,5	2,4	3,9	2,8	2,7	0,2	180
2,8	3	2,9	2,7	.	.	176
4,8	3,1	2,9	3,2	1,5	1,2	171
9,7	3,7	3,6	3,7	1,9	.	117,3
5,5	4,2	3,9	4,4	.	.	93,1
3	9,2	3,2	3,4	.	.	105
3,8	3,1	4,1	4,3	1,1		87,2
4,9	5,7	3,5	5,7	.	.	80
22,5	4,9	3,4	2,6	2,7	.	115
2,4	2,4	2,6	2,9	.	.	166
15,1	6,1	4,5	2,8	.	.	100,3
3,5	4,6	2,9	2,9	1,9	,	160
4,6	4	3,6	2,5	.	.	112
3,3	3,8	2,3	2,8	.	.	114,3
11,6	11,7	3,3	1,9	2,1	.	150,3
4,1	4,5	3	3,7	.	.	144
5,8	5,6	3,5	2,3	1,4	.	.
3,1	3,7	3,7	3,6	.	.	75
6,3	4,6	2,8	3	1,3	.	156
3,2	4,9	5,2	5,3	.	.	98,2
3,6	5,4	4,7	5,5	.	.	92,3
4,8	8,8	4,5	4,1	1,1		115,8
5,8	5,7	4,6	4,2	1,2		116,7
4,3	5,3	6,7	5,9	.	.	127,8
5,4	6,5	4,2	4	2,5		189,8
5,9	5,2	3,7	3,7	1,1		133,9
4	4,6	4,4	4,6	.	.	96
5,9	4,9	4,8	4,7	1,4	.	123
11,4	5,6	3,4	3,7	.	.	42
9	9,8	4,7	3,7	.	.	130
4,6	5,3	3,9	4,6	.	.	74
5,1	5,7	5,1	3,7	.	.	113
9	5,5	3,5	4,5	.	.	95,8
3,4	3,8	4,2	3,6	.	.	110
4,2	4,3	3,5	3,3	1,9		159,5
5,3	4,5	3,4	3,8			94,4
5,5	4,8	3,4	3,2	1,6		176,7
4,1	5	4,3	3,8	.	.	82
3,9	3,5	.	.	.	.	.
.	.	.	.	.	.	180
2,8	4	4,5	4	.	.	77
5,1	6,2	5	5,5	.	.	103,2
4,5	2,5	2,9	3,4	.	.	132
20,2	7,6	2,3	2,7	2,7	.	93
3,6	3,4	4,1	3,9	.	.	113

alfa	beta	bleb	m_bleb	Db	Hb
.	.	.	.	.	.
.	.	0	0	.	.
47,9	19,1	.	.	.	.
.	.	1	1	3,4	2,6
.	.	0	0	0	0
.	.	0	0	.	.
.	.	.	.	.	.
.	.	1	0	3,5	3,2
.	.	0	0	.	.
.	.	0	0	.	.
.	.	0	0	0	0
.	.	1	0	1,5	1,5
.	.	0	0	.	.
.	.	0	0	.	.
109,6	84,9	0	0	0	0
.	.	1	0	2,8	1,2
73,4	70,8	0	0	.	.
.	.	0	0	.	.
.	.	0	0	.	.
.	.	1	1	4,2	1,8
.	.	1	0	3	2
.	.	.	.	.	.
.	.	.	.	.	.
.	.	0	0	0	0
89,5	140,7	0	.	.	.
.	.	.	.	.	.
.	.	0	0	.	.
.	.	0	0	.	.
.	.	1	0	8,4	6,8
.	.	1	0	3,2	2,7
.	.	0	0	.	.
.	.	0	0	0	0
.	.	0	0	0	0
.	.	0	0	0	0
.	.	.	.	.	.
.	.	.	.	.	.
.	.	.	.	.	.
.	.	0	0	.	.
.	.	.	.	.	.
.	.	.	.	.	.
.	.	0	0	.	.
.	.	0	0	.	.
.	.	0	0	.	.
.	.	0	0	.	.
.	.	0	0	.	.
