# Supplementary material for: Intra-aneurysmal pressure changes during stent-assisted coiling
Source: PLoS One. 2020 Jun 4;15(6):e0233981. doi: 10.1371/journal.pone.0233981 (PMC7272096; doi:10.1371/journal.pone.0233981)
Supplement: Attachment — Submitted filename: Response to Reviewers.docx [file pone.0233981.s002.docx]

Dear Editors and Reviewers,

Thank you for your letter and for the reviewers’ comments concerning our manuscript “Intra-aneurysmal pressure changes during stent-assisted coiling.”

Thank you for the valuable reviewer’s comments. The critique was very constructive and relevant. The comments provided significant guidance to our study. We have carefully studied your comments and have made the recommended corrections and revisions. We hope that our revised manuscript is acceptable after the revision.

Please find below our corrections, and the responses to the reviewer’s comments are as

follows:

Reviewer #1: “The authors aimed to examine aneurysm hemodynamics with intra-saccular pressure measurement, and compare the effects of coiling, stenting and stent-assisted coiling in proximal segments of intracranial circulation. The manuscript deserves consideration. The Discussion must be improved a little bit. You should discuss more widely, expanding this section, on the importance of your findings in the light of the current literature, and highlighting the clinical effects of your results.”

***Our response****: We expanded the discussion to highlight the clinical effect of our observations. Hopefully, it has improved. Please refer to lines: 232-243, 254-256, and 262-266.*

Reviewer #2: “I really appreciated this manuscript. There are good insight of physics and hemodynamics. Nevertheless, I think that one or two diagrams (provided as images) may help readers to better understand your findings”.

***Our response****: We appreciate kind comments. Necessary changes were made. Additional figures were introduced – fig. 3 and 4.*

Kind regards,

Authors
